# A multi-objective algorithm for virtual machine placement in cloud environments using a hybrid of particle swarm optimization and flower pollination optimization

**DOI:** 10.7717/peerj-cs.834

**Published:** 2022-01-12

**Authors:** Sara Mejahed, M Elshrkawey

**Affiliations:** Information System Department Faculty of Computers and Information, Suez Canal University, Suez Canal University, Ismailia, Egypt

**Keywords:** Cloud computing, Virtual machine placement, Multi-objectives, Particle swarm optimization, Flower pollination optimization

## Abstract

The demand for virtual machine requests has increased recently due to the growing number of users and applications. Therefore, virtual machine placement (VMP) is now critical for the provision of efficient resource management in cloud data centers. The VMP process considers the placement of a set of virtual machines onto a set of physical machines, in accordance with a set of criteria. The optimal solution for multi-objective VMP can be determined by using a fitness function that combines the objectives. This paper proposes a novel model to enhance the performance of the VMP decision-making process. Placement decisions are made based on a fitness function that combines three criteria: placement time, power consumption, and resource wastage. The proposed model aims to satisfy minimum values for the three objectives for placement onto all available physical machines. To optimize the VMP solution, the proposed fitness function was implemented using three optimization algorithms: particle swarm optimization with Lévy flight (PSOLF), flower pollination optimization (FPO), and a proposed hybrid algorithm (HPSOLF-FPO). Each algorithm was tested experimentally. The results of the comparative study between the three algorithms show that the hybrid algorithm has the strongest performance. Moreover, the proposed algorithm was tested against the bin packing best fit strategy. The results show that the proposed algorithm outperforms the best fit strategy in total server utilization.

## Introduction

Virtualization is one of the most significant technologies in cloud computing systems. It allows the distribution of required resources to multiple users *via* multiple virtual machines (VMs) (Singh, 2018). Cloud computing infrastructure incorporates a large number of data centers (DCs) that can communicate over the internet. Each DC holds a vast number of VMs that are hosted on different physical machines (PMs). Hence, each user request is examined and modeled to determine the VM resources required to host and execute the requested task (Rashid & Chaturvedi, 2019). The allocation of VMs onto PMs is known as virtual machine placement (VMP) (Alashaikh & Alanazi, 2019). Cloud service providers attempt to exploit VMP methods to maximize the number of VMs placed within each PM, therefore maximizing the number of VMs that a DC can host. As such, VMP is an important topic in the research area of cloud computing (Gupta & Pateriya, 2014). Several factors make VMP a complex problem. These include scalability with respect to users requests, PM heterogeneity, and resource multi-dimensionality (Ghobaei-Arani, Shamsi & Rahmanian, 2017). Hence, VMP methodology should account for the specific requirements of a given DC. The efficacy of a VMP methodology can be measured by power consumption, cost, resource utilization, and load balancing (Masdari, Nabavi & Ahmadi, 2016). Varied research has been undertaken on metaheuristic algorithms (Alboaneen, Tianfield & Zhang, 2016; Donyagard Vahed, Ghobaei-Arani & Souri, 2019) including particle swarm optimization (PSO), genetic algorithms (GA), ant colony optimization(ACO), and the flower pollination algorithm (FPA). These algorithms were employed to find the optimal PM among all possible PMs in a DC. Optimal selection may be undertaken according to the aforementioned objectives. Metaheuristic algorithms are more advanced than exact strategies, which require high computational costs (Kumaraswamy & Nair, 2019). Consequently, metaheuristic algorithms are the superior choice for VMP. This is because the placement problem can be treated as an optimization problem that evaluates the best solution for placement among all candidate solutions.

Generally, the optimization decision of each metaheuristic algorithm is made based on the proposed fitness function. Each fitness function is formulated from parameters that refer to the aforementioned objectives. Hence, each algorithm formulates the optimal solution based on the combined values of the parameters which form the appropriate fitness function, corresponding to the overall objective.

The fitness functions used in existing research consider objectives such as reduction in power consumption, maximization of resource utilization, cost minimization, and load balancing (Adamuthe, Pandharpatte & Thampi, 2013). They do not consider placement time: the time consumed by placing a VM onto a cloud server. Specifically, placement time is the difference in time between a VM being requested, and the VM being placed onto a PM. This is an important metric for cloud service providers and potential users. An increase in placement time can violate the service level agreement (Addya et al., 2015). Such an infringement can upset users due to the violation of their technical requirements. Hence, placement time should be considered to avoid any deficiencies caused by placement time exclusion.

This paper proposes a novel model to address the problem of VMP by generating an optimal solution through three objectives. These objectives are minimization of the total time required to place each VM onto an appropriate PM, reduction in power consumption of the PMs in the DC, and the minimization of wasted resources. Hence, a novel multi-objective fitness function is proposed that incorporates three parameters that represent these objectives. According to the importance of the three objectives, the intended fitness function is the sum of three parameters of equal weight.

The proposed fitness function is implemented using three algorithms: particle swarm optimization with Lévy flight (PSOLF), flower pollination optimization (FPO), and a proposed hybrid algorithm (HPSOLF-FPO). The particle swarm optimization (PSO) algorithm has the capability to find the optimal solution by searching the local neighborhood for the current best solutions. However, the algorithm can become trapped in local optima even through the evaluation of successive iterations. In addition, particle velocities decrease rapidly, and particle positions converge and search within the same limited area to find the optimal solution from the candidate solutions (Schmitt & Wanka, 2015). Thus, the deduced optimal solution may be imprecise, as further possible solutions that may include the precise optimal solution are excluded. The addition of Lévy flight to update particle velocities is not sufficient for the algorithm to avoid becoming trapped in local optima, as the particle velocities decrease after several iterations and the problem reoccurs (Qingxi & Xiaobo, 2016). In addition, experiments show that the merging of Lévy flight with PSO causes a large number of VMs to be allocated onto a single PM. Accordingly, this allocation leads to substantial congestion on the selected PM.

The FPO algorithm is a modern optimization method derived from the pollination behavior of flowers, and uses both local and global search. It explores the local neighborhood of each of the best solutions such that the search spaces are guaranteed to be explored more efficiently (Nabil, 2016). However, due to the nature of the random search mechanism, over multiple iterations FPO struggles to find a compromise between global and local search. This deficiency reduces the exploitation capability of the algorithm and as such it tends towards fast convergence which limits its ability to find the optimal solution. Hence, the independent implementation of FPO is inefficient (Hoang, Bui & Liao, 2016).

This paper proposes the HPSOLF-FPO algorithm to overcome the deficiencies of the independent implementations of the PSO and FPO algorithms. The HPSOLF-FPO algorithm is designed to combine the exploration capabilities of FPO with the exploitation capabilities of PSO. Using the exploration capabilities of FPO, HPSOLF-FPO can move out of any local optima. Using the exploitation capabilities of PSO, the optimal solution can be obtained by intensifying the search around the local neighborhoods of the current best solutions. The hybrid algorithm produces superior results to the separate implementations of PSO or FPO. Furthermore, this combination gives rise to a search mechanism and accuracy which improves the placement decisions for the VMs.

The paper is organized as follows. ‘Related Work’ discusses the related work of VMP in a cloud computing environment. ‘VMP System Model and Problem Formulation’ details the mathematical formulation and system model of the optimization problem. ‘The Implementation of the Proposed Fitness Function Using PSO, FPO and HPSOLF-FPO’ describes the proposed VMP optimization algorithms: PSO, FPO, and HPSOLF-FPO. ‘Simulation Evaluation’ demonstrates and discusses the simulations and experimental results. Finally, ‘Conclusion and Future Work’ summarizes and concludes the paper and discusses possible future works.

## Related work

The placement of VMs onto appropriate PMs is a critical topic in cloud computing. Diverse algorithms have been proposed to resolve the issues and challenges facing this problem. Each algorithm attempts to distribute VMs onto PMs according to specific objectives. These objectives represent reduced power consumption, increased resource utilization, load balancing, cost minimization, and reduced service level agreement penetration (Gahlawat & Sharma, 2014; Pires & Barán, 2015; Zhao, Zhou & Li, 2019; Silva Filho et al., 2018). Hence, according to the implemented objectives, VMP algorithms may be categorized into two principal approaches: single-objective algorithms and multi-objective algorithms. Single-objective algorithms are deployed using just one of the aforementioned objectives. Saeedi & Shirvani (2021) propose a resource skewness-aware VM consolidation based on the improved thermodynamic simulated annealing algorithm, and a system framework with different modules that allow VMP to be modeled as an integer linear programming problem. The algorithm outperforms two heuristics and two metaheuristics in minimization of the number of used servers and reduction in data center resource wastage. Multi-objective algorithms are implemented by combining a selection of the aforementioned objectives into a single fitness function. Farzai, Shirvani & Rabbani (2020) propose a hybrid multi-objective genetics-based optimization solution for VMP by considering three objectives: reduced power consumption, reduced resource wastage, and reduced bandwidth usage in consideration of the data center topology for co-hosting dependent VMs.

Generally, VMP algorithms may be classified into four principal classes (Attaoui & Sabir, 2018). These classes are heuristic, metaheuristic, exact, and approximate. Exact algorithms include constraint programming (Van, Tran & Menaud, 2010; Mann, 2016), integer linear programming (López, Kushik & Zeghlache, 2019), mixed integer linear programming (Regaieg et al., 2018), and pseudo-boolean optimization (Ribas et al., 2013). Although these algorithms generate optimal solutions, they suffer from exponential time complexity. Coffman et al. (2013) develop multiple approximate algorithms. Upon inspection, these algorithms are capable of resolving one-dimensional bin packing. However, it has been verified that two-dimensional bin packing algorithms cannot be examined in polynomial time. It is therefore unlikely that an exact algorithm or an effective approximate algorithm is suitable (Mann, 2015).

In consideration of this, recent research has been directed towards heuristic or metaheuristic algorithms (Mollamotalebi & Hajireza, 2017; Chang et al., 2018; Satpathy et al., 2018; Masdari et al., 2019). The first fit decreasing algorithm (Keller et al., 2012) places the PMs into a successive list and sorts the VMs in descending order according to their resource demand. When a VM is hosted, it selects the first PM that has adequate resources. The best fit decreasing algorithm (Varasteh & Goudarzi, 2015) imitates the first fit decreasing algorithm by placing the VMs in descending order. Subsequently, the VM is allocated to a PM which has the minimum remaining resources adequate for this VM. The modified best fit decreasing algorithm (Esfandiarpoor, Pahlavan & Goudarzi, 2015) arranges the VMs according to their CPU requirement. Following this, a VM is assigned to the server which produces the smallest energy increment for the DC. For this reason, the preferred server is selected from the servers that are neither empty nor fully utilized. The objective of this algorithm is the minimization of power consumption by the DC. However, it does not consider the increase in resource utilization. The integer quadratic program with linear and quadratic constraints (Vakilinia, 2018) aims to optimize power in the DC. Additionally, server power consumption, migration cost, and network communication *via* VMP are optimized. However, the approach is not consistently successful as the system scales up in size. The resource aware VMP algorithm (Gupta & Amgoth, 2018) aims to minimize the number of active PMs to reduce power consumption and resource wastage. The approach uses a new concept: resource usage factor. This is used to balance resource optimization on active PMs. The algorithm performs no operations on the VM and PM lists, but instead computes the resource usage factor of each PM according to the requirements of the VM resource. Following this, the calculated factor is used to select a suitable PM for the VM such that resources are optimized. Although this approach maximizes resource optimization and reduces resource wastage, it does not consider the power consumed. The GA proposed by Jamali & Malektaji (2014) is based on the vector packing approach, and aims to minimize power consumption by maximizing resource usage and reducing the number of active PMs. The algorithm successfully reduces power consumption, however resources are used inefficiently and reduction in resource wastage is not accomplished.

The improved Lévy-based whale optimization algorithm presented by Abdel-Basset, Abdle-Fatah & Sangaiah (2019) is used to allocate VMs according to the current cloud computing bandwidth. The proposed approach generates an optimal balance for network load. However, the initial placement of VMs is not a feasible approach to load balancing due to the continuous increase in task number and cloud size. Recently, an approach based on an ant colony system (Alharbi et al., 2019) was proposed for dynamic VMP to minimize the power consumption in DCs. The approach uses a novel heuristic such that the PM with minimum power usage is designated to host VMs. This reduces DC power consumption but leads to a long execution time which is problematic for large-scale DCs. Furthermore, the effect on resource wastage was not measured. A VMP approach based on multi-cloud flower pollination optimization (Usman et al., 2018) aims to maximize resource utilization and reduce DC power consumption. The approach supports the use of clustering, migrations, and power effectiveness techniques. However, implementations of the approach have not successfully maximized resource utilization for the multi-cloud. The framework introduced by Usman et al. (2019) obtains optimal VM placements onto appropriate PMs by applying a flower pollination optimization algorithm. The approach uses a strategy known as dynamic switching probability, which aims to efficiently acquire an optimal placement solution and improves the performance of cloud DCs. However, this strategy scales poorly. Fatima et al. (2018) resolve the VMP problem by merging an improved Lévy-based particle swarm optimization algorithm and a variable-sized bin packing algorithm that uses the best-fit strategy. The method initializes with the standard operations of basic PSO: the swarm is applied in research space, and local and global best solutions are predicted. The remaining steps are guided based on a probability value. If the probability value is greater than 0.5, a basic PSO method is used to update the particle velocities. Otherwise, the particle velocities are updated by Lévy flight. However, this method tends to allocate a large number of VMs onto a single PM which causes substantial congestion on the selected PM. The improved PSO approach of Jensi & Jiji (2016) exploits Lévy flight to obtain new particle velocities. The proposed algorithm has been verified by 21 well-known test functions, and is shown to improve the aptitude of comprehensive search and increase the efficiency of convergence. However, the algorithm is limited to linear problems.

A hybrid genetic wind-driven algorithm is proposed by Javaid et al. (2017). A controller is inserted into a smart grid to manage energy for a residential area by use of a heuristic algorithm that balances the load in the grid area network. The approach is applicable to single or multiple homes, although a high request rate introduces delay. The discrete three-phase hybrid PSO algorithm proposed by Shirvani (2020) solves parallelizable scheduling on heterogeneous computing systems. The proposed algorithm merges discrete PSO with the hill climbing technique to avoid the local optima problem. In Kumar & Mandal (2017), a cloud model of VMP is simulated using three optimization algorithms: PSO, GA, and hybrid GA PSO. The proposed model reduces the number of active physical servers, lessens power consumption, and reduces cloud resource wastage. However, the proposed model lacks the means to update the velocity and position of each particle. This capability is necessary to make long jumps toward an optimal solution. In addition, the proposed model requires substantial adjustments to achieve load balance.

## VMP system model and problem formulation

This section demonstrates the VMP prefaces and applied architecture. In addition, the problem formulation is presented. Moreover, the proposed fitness function, which is central to the implementation of the proposed optimization algorithms, is presented. The fitness function is a multi-objective function which is designed to obtain the optimal values for placement time, power consumption, and resource wastage of the PMs within the cloud DC.

### VMP model

**Figure 1:**
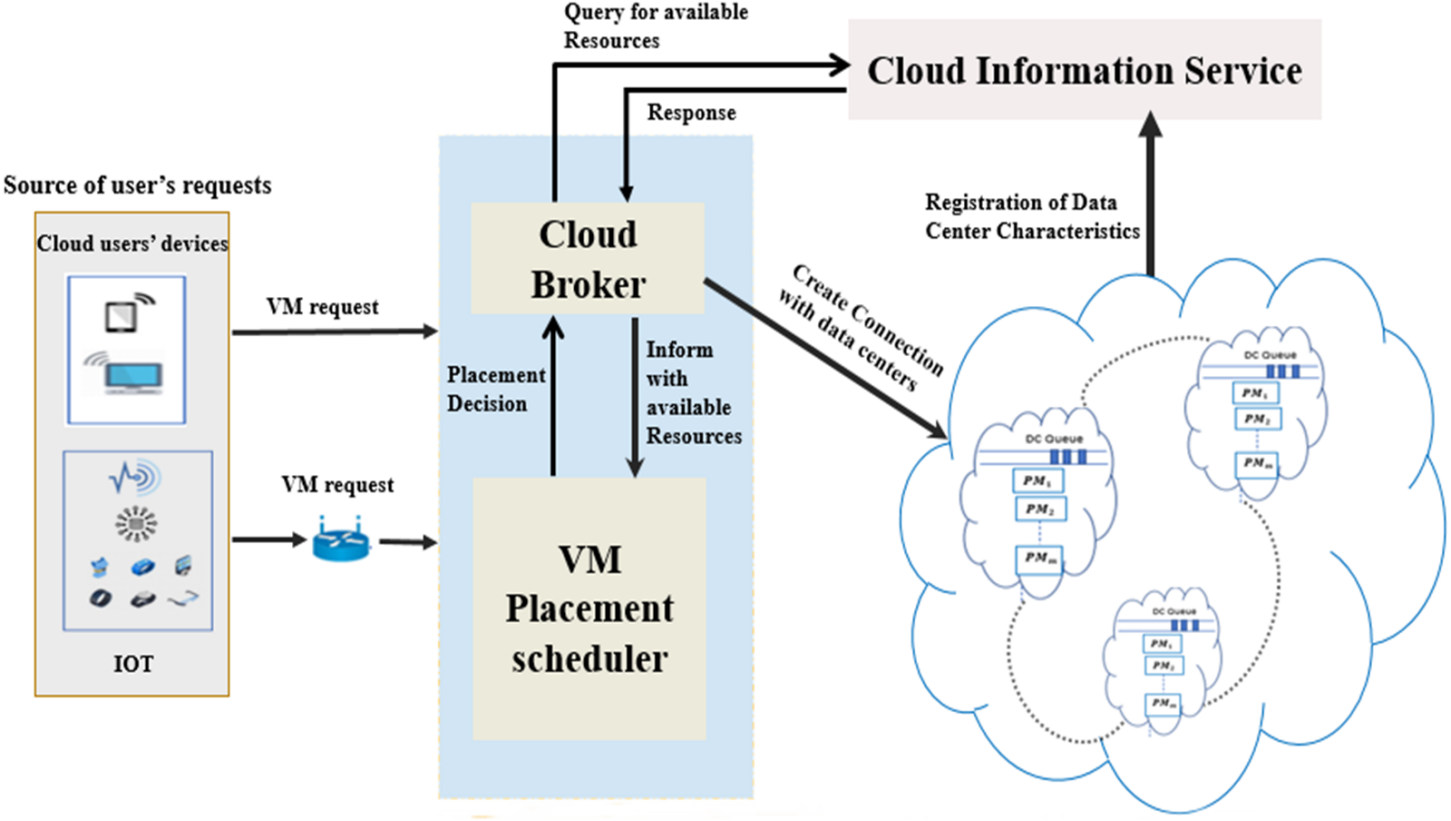
The system model of VMP in cloud DCs.

The architecture of the implemented VMP model is depicted in Fig. 1. It comprises four key components: the cloud DCs, cloud information service, system mediator (including cloud broker and VMP scheduler), and the cloud users’ devices. The cloud information service registers the status of each PM within the cloud DCs. In addition, it continuously notifies the cloud broker of every status update concerning the PMs that have adequate resources to host requested VMs. The status should include the service rate offered by the PM in the DC, and the expected waiting time in the queue of each DC.

 Consequently, the cloud broker has two roles. First, it receives all user VM requests. Second, it converts the gathered information concerning available PMs and requested VMs into apposite vector forms and submits them to the VMP scheduler. The scheduler uses these vectors to make the placement decisions for the requested VMs using the proposed fitness function and optimization algorithms. In accordance with the scheduler decisions, the requested VMs are placed onto the appropriate PMs.

### Problem formulation

To streamline the formulation of the VMP model, the mathematical formulation of the VMP problem is as follows. Let *P* = {*p*_1_, *p*_2_, *p*_3_, …, *p*_*N*_} be a set of *N* available PMs within a given DC. We assume that the resource capacity of *p*_*i*_ is completely defined by its CPU and memory. Let *C* = {*c*_1_, *c*_2_, *c*_3_, …, *c*_*N*_} and *M* = {*m*_1_, *m*_2_, *m*_3_, …, *m*_*N*_} be two sets denoting respectively the CPU and memory resources of the PMs. Let *V* = {*v*_1_, *v*_2_, *v*_3_, …, *v*_*M*_} be a vector of *M* requested VMs. In addition, the CPU and memory requirements of these VMs can be expressed respectively as *C*′ = $\{ {c}_{1}^{{}^{{^{\prime}}}},{c}_{2}^{{}^{{^{\prime}}}},{c}_{3}^{{}^{{^{\prime}}}},\ldots ,{c}_{M}^{{}^{{^{\prime}}}}\} $ and *M*′ = $\{ {m}_{1}^{{}^{{^{\prime}}}},{m}_{2}^{{}^{{^{\prime}}}},{m}_{3}^{{}^{{^{\prime}}}},\ldots ,{m}_{M}^{{}^{{^{\prime}}}}\} $. To establish the mapping between the *M* requested VMs and the *N* available PMs, an *N* × *M* placement matrix 𝕄, which defines the possible assignments of the mapping, is defined as follows:

(1) \begin{eqnarray*}\mathbb{M}={P}^{T}\times V,\end{eqnarray*}

where *P*^*T*^ is the transpose of the row vector *P*. Each element *e*_*ij*_ = *p*_*i*_ *v*_*j*_ ∈ 𝕄 defines a possible placement for each VM *v*_*j*_ on a single PM *p*_*i*_, 1 ≤ i ≤ *N*, 1 ≤ j ≤ *M*. Each VM may have possible placements on multiple PMs. Hence, the decision binary variable *e*_*ij*_ can be precisely identified as:

(2) \begin{eqnarray*}{e}_{ij}= \left\{ \begin{array}{@{}ll@{}} \displaystyle 1, &\displaystyle \text{if}~{v}_{j}~\text{placed on}{p}_{i}\\ \displaystyle 0, &\displaystyle \text{otherwise.} \end{array} \right. \end{eqnarray*}

Let us define *X* to be the vector of PMs that have a probability to host at least one VM. Each decision variable *x*_*k*_ ∈*X*, 1 ≤ k ≤ *N* can be expressed as:

(3) \begin{eqnarray*}{x}_{k}= \left\{ \begin{array}{@{}ll@{}} \displaystyle 1, &\displaystyle \sum _{j=1}^{M}{e}_{kj}\geq 1\\ \displaystyle 0, &\displaystyle \text{otherwise.} \end{array} \right. \end{eqnarray*}

However, the values of each element *e*_*ij*_ ∈ 𝕄 and *x*_*k*_ ∈ *X* are set to a binary decision variable which equals 1 if and only if the following two constraints are fulfilled:

(4) \begin{eqnarray*} \frac{{C}^{{}^{{^{\prime}}}}}{C} \leq 1\end{eqnarray*}

and

(5) \begin{eqnarray*} \frac{{M}^{{}^{{^{\prime}}}}}{M} \leq 1.\end{eqnarray*}

The normalized CPU utilization ${U}_{i}^{cpu}$ offered by the PM *p*_*i*_ to all hosted VMs can be defined as follows:

(6) \begin{eqnarray*}{U}_{i}^{cpu}=\sum _{j=1}^{M} \frac{{e}_{ij}{c}_{j}^{{}^{{^{\prime}}}}}{{c}_{i}} .\end{eqnarray*}

The normalized RAM utilization ${U}_{i}^{ram}$ offered by the PM *p*_*i*_ to all hosted VMs can be defined as follows:

(7) \begin{eqnarray*}{U}_{i}^{ram}=\sum _{j=1}^{M} \frac{{e}_{ij}{m}_{j}^{{}^{{^{\prime}}}}}{{m}_{i}} .\end{eqnarray*}

The role of the VMP scheduler is to obtain the optimal placement solution with respect to three objectives: minimization of placement time, minimization of power consumption, and minimization of resource wastage. The following subsections introduce the mathematical formulas for these three components.

#### Modeling placement time

This subsection derives a mathematical expression for the placement time *T*_*j*_ of a VM *v*_*j*_. The placement time *T*_*j*_ is measured from the instant at which *v*_*j*_ is requested to the expected instant at which *v*_*j*_ is to be placed onto a specified PM *p*_*i*_. From this definition, *T*_*j*_ consists of two components.

1. The search time *S*_*j*_ is the time required by *v*_*j*_ to find all the available PMs *x*_*k*_ ∈*X* that fulfill its requirements, where |*X*| = *N*. Hence, the value of *S*_*j*_ can be written as
  (8) \begin{eqnarray*}{S}_{j}=\sum _{k=1}^{N}{x}_{k} \left( \right. T \left( \right. \frac{{c}_{j}^{{}^{{^{\prime}}}}}{{c}_{k}} + \frac{{m}_{j}^{{}^{{^{\prime}}}}}{{m}_{k}} \left( \right. +{\delta }_{k} \left( \right. ,\end{eqnarray*}
  where *T* is the time required to find appropriate PMs on which to place the requested VMs, and *δ*_*k*_ is the search time factor representing the time delay for each VM cycle.
2. The expected waiting time *E*[*W*_*j*_] of *v*_*j*_ in the cloud DC consists of the VM queuing time and the VM service time. Let the arrival process of a given VM at the cloud DC be a Poisson process with arrival rate *λ*. Let the service time of a VM be exponentially distributed with expectation $ \frac{1}{\mu } $. We assume that the DC has an infinite queue for the hosting of all arrived VMs on *N* PMs. From this physical description, the system dynamics (*i.e.,* arrival, service, and departure) can be modeled as the well-known multi-server queuing system M/M/N, where *λ* < *μ* is the steady state condition. The expected waiting time *E*[*W*_*j*_] of M/M/N can be written as (White, 2012)
  (9) \begin{eqnarray*}E[{W}_{j}]= \frac{\mu \left( \right. \frac{\lambda }{\mu } { \left( \right. }^{N}{p}_{0}}{(N-1){!}(N\mu -\lambda )^{2}} + \frac{1}{\mu } ,\end{eqnarray*}
  where *p*_0_ is the probability of an empty queue and is given by
  (10) \begin{eqnarray*}{p}_{0}= \left[ \right. \sum _{n=0}^{N-1} \frac{( \frac{\lambda }{\mu } )^{n}}{n} + \frac{( \frac{\lambda }{\mu } )^{n}}{N{!}(1- \frac{\lambda }{N\mu } )} { \left( \right. }^{-1}.\end{eqnarray*}

Using Equations Eqs. (8) and (9), the placement time *t*_*j*_ of *v*_*j*_ is given as

(11) \begin{eqnarray*}{t}_{j}={S}_{j}+E[{W}_{j}].\end{eqnarray*}

The total placement time *T*_*j*_ can be calculated as follows:

(12) \begin{eqnarray*}{T}_{j}=\sum _{i=0}^{N}{e}_{ij}{t}_{j}.\end{eqnarray*}

Finally, the total placement time *T* for all VMs can be calculated using Eq. (12):

(13) \begin{eqnarray*}T=\sum _{j=0}^{M}{T}_{j}.\end{eqnarray*}

#### Modeling power consumption

Jin et al. (2020) accurately modeled power consumption linearly as a function of the resource utilization of the PMs in the cloud DC. In this model, power consumption is based only on CPU utilization. Additionally, unused PMs are deactivated to save power. Hence, power consumption can be computed for each PM *p*_*i*_ ∈ *P* as follows. Let ${u}_{i}^{max}$ and ${u}_{i}^{idle}$ be the power consumption of PM *p*_*i*_ at maximum CPU utilization and idle state, respectively. The power consumption *PU*_*i*_ of the PM *p*_*i*_ due to the VM *v*_*j*_ is given as

(14) \begin{eqnarray*}P{U}_{i}={u}_{i}^{idle}+ \left( \right. {u}_{i}^{max}-{u}_{i}^{idle} \left( \right. \frac{{e}_{ij}{c}_{j}^{{}^{{^{\prime}}}}}{{c}_{i}} .\end{eqnarray*}

The power consumption $P{U}_{i}^{{}^{{^{\prime}}}}$ of the PM *p*_*i*_ due to all VMs is given using Equation (6) as

(15) \begin{eqnarray*}P{U}_{i}^{{}^{{^{\prime}}}}={u}_{i}^{idle}+ \left( \right. {u}_{i}^{max}-{u}_{i}^{idle} \left( \right. {U}_{i}^{cpu}.\end{eqnarray*}

From Eqs. (14) and (15), the total power consumption *PU* of all PMs in a DC can be computed as follows:

(16) \begin{eqnarray*}PU=\sum _{i=0}^{N}{x}_{i}\times P{U}_{i}^{{}^{{^{\prime}}}}.\end{eqnarray*}

#### Modeling resource wastage

The residual resources offered by each physical machine may vary according to the VMP strategy. To maximize the utilization of multiple resources such as CPU and RAM, the resource wastage *W*_*i*_ of the PM *p*_*i*_ can be calculated as follows:

(17) \begin{eqnarray*}{W}_{i}= \frac{ \left\vert \right. {r}_{i}^{cpu}-{r}_{i}^{ram} \left\vert \right. }{{U}_{i}^{cpu}+{U}_{i}^{ram}} +,\end{eqnarray*}

where ${r}_{i}^{cpu}$ and ${r}_{i}^{ram}$ represent the normalized residual CPU and memory resources; the values of ${U}_{i}^{cpu}$ and ${U}_{i}^{ram}$ are given in Eqs. (6) and (7); and *ɛ* = 0.001, a small positive real number (Gupta & Amgoth, 2016). This model and its corresponding objective are included to make best use of the resources of all PMs and achieve balance between multiple residual resources. The total resource wastage *W* of all PMs in a DC is given by

(18) \begin{eqnarray*}W=\sum _{i=1}^{N}{x}_{i} \frac{ \left\vert \right. \left( \right. {U}_{i}^{cpu}-\sum _{j=1}^{M}{e}_{i,j}{c}_{j}^{{}^{{^{\prime}}}} \left( \right. - \left( \right. {U}_{i}^{ram}-\sum _{j=1}^{M}{e}_{i,j}{m}_{j}^{{}^{{^{\prime}}}} \left( \right. \left\vert \right. +}{\sum _{j=1}^{M}{e}_{i,j}{c}_{j}^{{}^{{^{\prime}}}}+\sum _{j=1}^{M}{e}_{i,j}{m}_{j}^{{}^{{^{\prime}}}}} .\end{eqnarray*}

Finally, the placement problem can be formulated:

(19) \begin{eqnarray*}Minimize~~T.\end{eqnarray*}

(20) \begin{eqnarray*}Minimize~~PU.\end{eqnarray*}

(21) \begin{eqnarray*}Minimize~~W.\end{eqnarray*}

Subject to the constraints

(22) \begin{eqnarray*}\sum _{i=1}^{N}{e}_{i,j}=1, \forall j=1,2,\ldots ,M,\end{eqnarray*}

(23) \begin{eqnarray*}\sum _{j=1}^{M}{e}_{i,j}{c}_{j}^{{}^{{^{\prime}}}}\lt {c}_{i}, \forall i=1,2,\ldots ,N,\end{eqnarray*}

(24) \begin{eqnarray*}\sum _{j=1}^{M}{e}_{i,j}{m}_{j}^{{}^{{^{\prime}}}}\lt {m}_{i}, \forall i=1,2,\ldots ,M,\end{eqnarray*}

and

(25) \begin{eqnarray*}{x}_{i},{e}_{i,j}\in \{ 0,1\} , \forall i=1,2,\ldots ,N,j=1,2,\ldots ,M.\end{eqnarray*}

From Eq. (22), each VM can be placed only on a single PM. Equations (23) and (24) stipulate that the summation of CPU and RAM respectively of all VMs hosted on a given PM must not surpass that PMs CPU and RAM capacity. Finally, Eq. (25) formalizes the domains of the variables *M* and *N*. Hence, there are *M*^*N*^ possible solutions for the placement problem.

### The fitness function

The primary goal of this work is to obtain the optimal solution for VMP. This is accomplished by the use of a fitness function, which allows the optimizer to select the optimal solution as a function of the three objectives described in Eqs. (19)–(21). The fitness function is created from the three objective functions by using the scalarization method (Gunantara, 2018):

(26) \begin{eqnarray*}F(x)={w}_{1}{f}_{1}(x)+{w}_{2}{f}_{2}(x)+\cdots +{w}_{n}{f}_{n}(x),\end{eqnarray*}

where *F*(*x*) is the total fitness function and *w*_1_, *w*_2_, …, *w*_*n*_ represent the weight values given to each of the objective functions. Each weight value is determined by the priority of its corresponding objective function within the total fitness function. The weight value has a vital role in governing the performance of the corresponding objective function within the total fitness function (Giagkiozis & Fleming, 2015). Hence, the weight values should be determined prior to optimization. In this paper, equal weighting is assigned to each component of the fitness function. This approach is used to equalize priorities for each objective: minimization of placement time, minimization of power consumption, and minimization of resource wastage.In this case each weight can be calculated as *w*_*i*_ = 1/*n*, where *n* is the number of objective functions. Accordingly, the equal weight value of each objective function is *w*_*i*_ = 1/3.

## The implementation of the proposed fitness function using PSO, FPO and HPSOLF-FPO

This section presents the proposed nature-inspired algorithms for cloud computing VMP: PSO, FPO, and PSOLF-FPO. These algorithms use the proposed fitness function to find the optimal placements for a set of VMs on available PMs within a cloud DC while minimizing total placement time, total power consumption, and resource wastage.

### The PSO algorithm

The PSO algorithm is modeled on the social behavior of fish and bird swarms (Clerc, 2010). The following subsections describe the creation and configuration of the set of particles that embody the swarm. In addition, the parameters of PSO are described in detail.

#### Particle encoding

Each particle represents a candidate solution for the placement of a set of VM requests on a set of PMs. The placement matrix 𝕄, defined in Eq. (1), is used to initiate the particles. In this manner, each created particle represents one of the *M*^*N*^ possible solutions to the placement problem.

**Figure 2:**
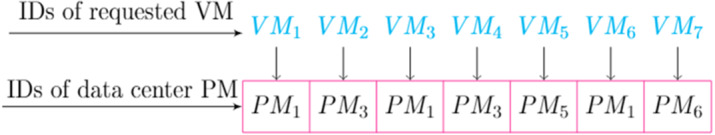
An example of particle encoding.

For example, consider two different particles created from 𝕄 in the initial swarm as shown in Fig. 2. Each particle is created in a two-dimensional scheme that uses one-to-many maps between each PM and its hosted VMs within the particle.

#### Particle evaluation

The optimal solution for placement is obtained through successive iterations of swarm regeneration. A new swarm is generated whenever the position and velocity of the particles are updated. The equations for particle position and velocity as implemented in the standard PSO and PSOLF algorithms are as follows. Using PSO, the velocity of each particle is updated as

(27) \begin{eqnarray*}{V}_{i}(t+1)=\omega \times {V}_{i}(t)+{\rho }_{1}\times {c}_{1}\times ({P}_{lbest}-{P}_{i}(t))+{\rho }_{2}\times {c}_{2}\times ({P}_{gbest}-{P}_{i}(t)),\end{eqnarray*}

where *i* is the particle number within the swarm, *ω* is the inertia weight which determines the impact of the previous velocity on the current velocity, *V*_*i*_(t) is the current particle velocity, *V*_*i*_(t + 1) is the new particle velocity, *P*_*i*_(t) is the current position of the particle within the swarm, *C*_1_ and *C*_2_ are learning factors of the particle and swarm, and *ρ*_1_ and *ρ*_2_ are uniformly distributed random variables between 0 and 1. Using PSO, the position of each particle is updated as

(28) \begin{eqnarray*}{P}_{i}(t+1)={P}_{i}(t)+{V}_{i}(t+1),\end{eqnarray*}

where *P*_*i*_(*t* + 1) is the new position of the particle within the search space, and *P*_*i*_(*t*) is the current position. Standard PSO has a defect known as premature convergence that pushes the particles to converge too early and become trapped within local optima (Nakisa et al., 2014). To overcome this problem, Lévy flight can be combined with PSO to produce PSOLF. This reduces early particle convergence by updating velocities in a manner that causes the particles to take a long step towards the optimal solution (Haklı& Uğuz, 2014). When using Lévy flight, the particle positions and velocities are updated as follows. Using PSOLF, the velocity of each particle is updated as (Jensi & Jiji, 2016)

(29) \begin{eqnarray*}{V}_{i}(t+1)=\omega \times L\times {V}_{i}(t)+{\rho }_{1}\times {c}_{1}\times ({P}_{lbest}-{P}_{i}(t))+{\rho }_{2}\times {c}_{2}\times ({P}_{gbest}-{P}_{i}(t)),\end{eqnarray*}

where *L* is a step size emulating the large distance. This can be calculated using Lévy flight:

(30) \begin{eqnarray*}L(s,a)\sim \frac{\lambda \times \Gamma (\lambda )\times \sin \nolimits \frac{(\pi \lambda )}{2} }{\pi } ,\end{eqnarray*}

where Γ(*λ*) is the gamma function with index *λ*, *a* = 1 is a control parameter of the distribution, and *S* is a large step which is given by

(31) \begin{eqnarray*}S= \frac{U}{{|}V{{|}}^{(\lambda -1)}} .\end{eqnarray*}

The parameters *U* and *V* are drawn from a Gaussian normal distribution and are given by

(32) \begin{eqnarray*}U\sim N(0,{\sigma }_{u}^{2}),V\sim N(0,{\sigma }_{v}^{2}),\end{eqnarray*}

where

(33) \begin{eqnarray*}{\sigma }_{u}={ \left[ \frac{\Gamma (1+\lambda )}{\lambda \Gamma ( \frac{(1+\lambda )}{2} )} \times \frac{\sin \nolimits ( \frac{\pi \lambda }{2} )}{{2}^{ \frac{(\lambda -1)}{2} }} \right] }^{ \frac{1}{\lambda } }.\end{eqnarray*}

The size of each step is calculated using Jensi & Jiji (2016)

(34) \begin{eqnarray*}stepsize=0.01\ast S.\end{eqnarray*}

Using PSOLF, the position of each particle is updated as

(35) \begin{eqnarray*}{P}_{i}(t+1)={V}_{i}(t+1).\end{eqnarray*}

#### The PSOLF-based VMP algorithm stages

Before describing the proposed PSOLF-based VMP algorithm, there are some preparatory PSO principles that should be clarified and described. In the search space, each particle has position *P*_*i*_ and velocity *V*_*i*_. Both should be computed at each iteration of the swarm evaluation. In addition, each particle has a key criterion known as the local best solution *P*_*l*−*best*_. During each iteration, *P*_*l*−*best*_ is updated with the particles computed position value if the computed value is less than *P*_*l*−*best*_. The swarm itself has an analogous criterion known as the global best solution *P*_*g*−*best*_. Generally, the global best solution is given by the minimum value of the local best solution among all particles.

The proposed algorithm consists of three principal operations: swarm initialization, swarm evaluation, and termination.

• **Swarm initialization.** The swarm is initialized by generating a particle vector *P* from the placement matrix 𝕄. All initiated particles are restricted by the constraints in Eqs. (22)–(25). The number of particles in the swarm is *N*_*swarm*_. For each particle, the initial position and velocity are generated randomly based on the particles index within the vector *P*. The position of each particle is set to its corresponding local best solution *P*_*l*−*best*_. The minimum value of *P*_*l*−*best*_ among all particles is assigned to the global best *P*_*g*−*best*_ of the swarm. In addition, the required PSO parameters are defined, including the learning factors *c*_1_, *c*_2_, inertia weight coefficient *ω*, and the random variables *ρ*_1_, *ρ*_2_ ∈ [0,1]. Moreover, the random function *rand*() is defined to generate random numbers between [0,1], and the maximum number of iterations is set to *Max*_*iter*_. Finally, the fitness function of all particles is computed using Eq. (26) to obtain the local fitness of each particle.
• **Swarm evaluation.** During each iteration, the particle velocities and positions are updated by either PSO or PSOLF. The choice between algorithms is dependent upon the value generated by *rand*(). If the value of *rand*() is less than 0.5, the particle velocities and positions are updated as in Eqs. (29) and (35). Else, the particle velocities and positions are updated as in Eqs. (27) and (28). Subsequently, the fitness values associated with the updated particle position *P*_*u*_ and the position *P*_*l*−*best*_ are compared. If the fitness value of *P*_*u*_ is less than the fitness value of *P*_*l*−*best*_, then *P*_*l*−*best*_ is set to *P*_*u*_. Furthermore, if the fitness value of *P*_*l*−*best*_ is less than the fitness value of the global best position *P*_*g*−*best*_, then *P*_*g*−*best*_ is set to *P*_*l*−*best*_.
• **Swarm termination.** Swarm evaluation continues iteratively until the maximum number of iterations *Max*_*iter*_ is reached. Following the final iteration, the particle corresponding to *P*_*g*−*best*_ is selected to represent the optimal fitness value for the VMP solution.

The pseudo-code for the PSOLF algorithm is presented below.


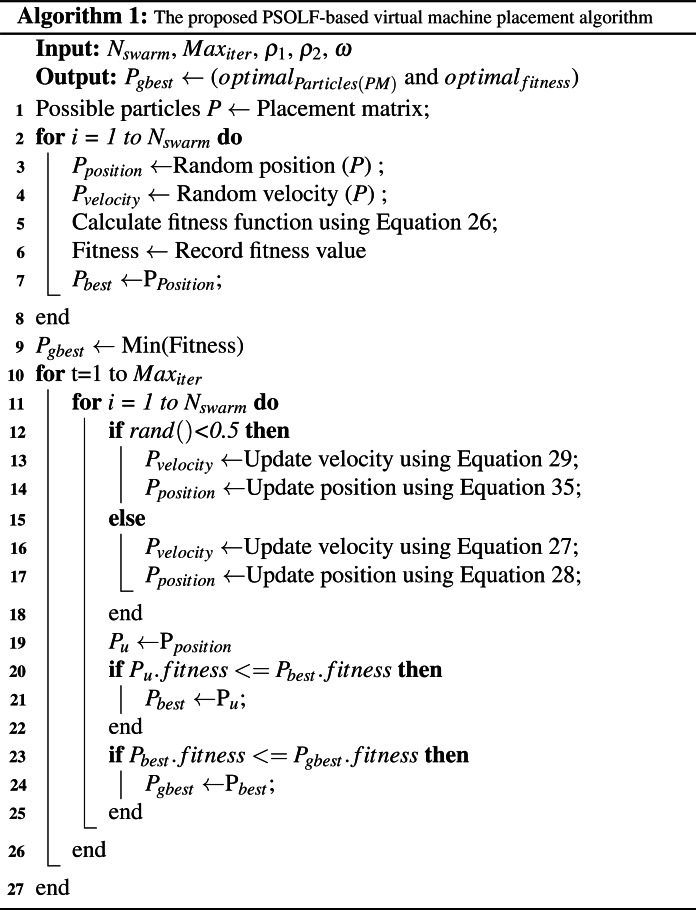


### The FPO algorithm

Developed by Yang (2012), FPO is a fascinating algorithm based on the process of flower pollination in flowering plants. It employs features of the pollination process to evaluate both global pollination and local pollination (Yang, Karamanoglu & He, 2014).

#### Pollen encoding

**Figure 3:**
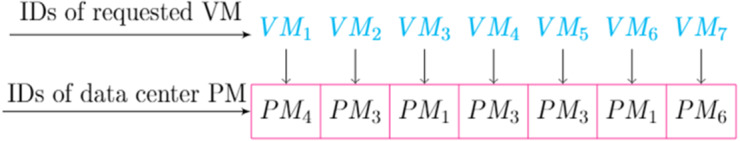
An example of pollen encoding.

Each pollen grain encodes a possible VMP solution. The placement matrix 𝕄 can be used to initialize the pollen as in the case of the particle swarm. An example of the pollen initialization is shown in Fig. 3. Each pollen grain is created in a two-dimensional scheme that uses one-to-many maps between each PM and its hosted VMs within the pollen grain.

#### Pollen evaluation

The two principal steps of the FPO algorithm are represented by two pollination methods: global pollination and local pollination. The method used is selected based on a switching probability value *β*. The optimal placement solution is obtained through a series of iterations. During each iteration a random number between [0,1] is generated. This number is compared with the switch probability *β*. If the random number is less than *β*, global pollination will be used and updated using the Lévy distribution. Else, local pollination is used. When using global pollination, the pollen vectors are updated as

(36) \begin{eqnarray*}{S}_{i}^{(t+1)}={S}_{i}^{t}+L\times ({S}_{i}^{t}-{g}_{best}^{\ast }),\end{eqnarray*}

where ${S}_{i}^{t}$ is the *i*th pollen vector (solution) at iteration *t*, ${g}_{best}^{\ast }$ is the global best solution at the current iteration, and *L* is the pollination strength and is calculated in the same manner as the step size using Eqs. (30)–(34). When using local pollination, the pollen vectors are updated as

(37) \begin{eqnarray*}{S}_{i}^{(t+1)}={S}_{i}^{t}+[{S}_{r1}^{t}-{S}_{r2}^{t}],\end{eqnarray*}

where ${S}_{r1}^{t}$ and ${S}_{r2}^{t}$ are a random selection of pollen grains (solutions) for local pollination.

#### The FPO-based VMP algorithm stages

The FPO algorithm consists of three principal operations: pollen initialization, population evaluation, and termination.

• **Pollen initialization.** To initialize the pollen, the placement matrix 𝕄 is used to generate all possible placement solutions *S*_*i*_. All solutions must obey the placement constraints in Eqs. (22)–(25). Each solution *S*_*i*_ is assigned randomly to a pollen grain. In addition, the fitness function for each pollen grain is computed using Eq. (26). The minimum fitness value among the pollen is selected as ${g}_{best}^{\ast }$. Furthermore, the population size is set to *N*_*pop*_, and the maximum number of iterations is set to *N*_*iter*_. Finally, the switching probability is defined as *β* ∈ [0,1].
• **Population evaluation.** During each iteration a random number ∈ [0,1] is generated and compared with the switching probability *β*. If the random number is less than *β* then global pollination and Eq. (36) are used. Else, local pollination and Eq. (37) are used. Subsequently, the results of the new solutions *S*_*u*_ are evaluated using the pre-computed fitness functions. If the fitness value of the new solution is less than the fitness value of ${g}_{best}^{\ast }$, then ${g}_{best}^{\ast }$ is set to the new solution.
• **Termination.** Population evaluation continues iteratively until the maximum number of iterations *N*_*iter*_ is reached. Following the final iteration, ${g}_{best}^{\ast }$ is selected as the optimal VMP solution.

The pseudo-code for the FPO algorithm is presented below.


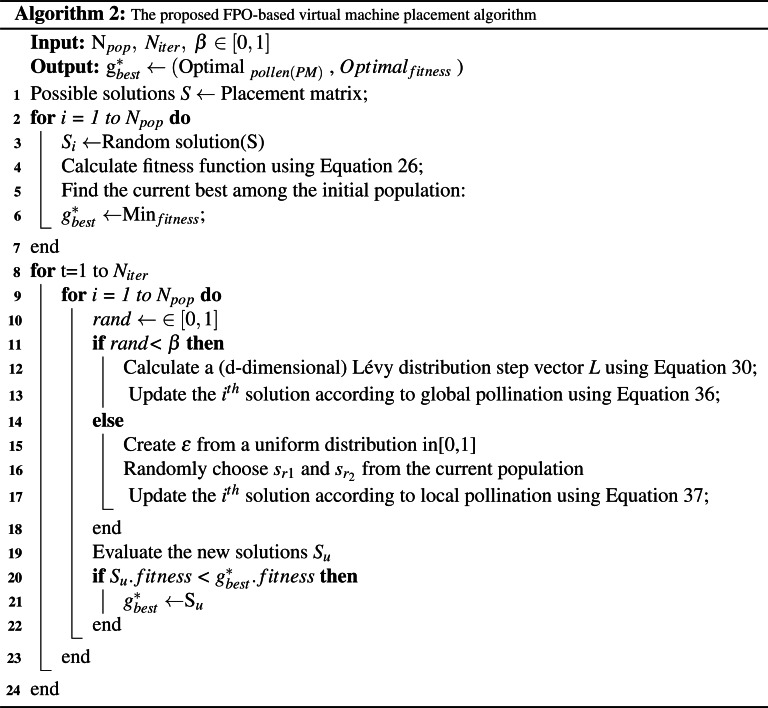


### The hybrid PSOLF-FPO algorithm

The trap of local optima is the primary obstacle to the use of PSO. This problem causes particle velocities to decrease quickly over successive iterations. Consequently, the particle positions will converge and search the local area of the same peak. Thus, the resulting solution may not be optimal, due to the exclusion of other possible solutions further away from the local peak. Despite the use of Lévy flight to update the particle velocities (Hariya et al., 2015), the problem remains, as the Lévy flight implementation ensures that the particle velocities will decrease after several iterations. Consequently, the local optima may disappear for several iterations before reappearing. In addition, the PSOLF algorithm tends to allocate a large number of VMs onto a single PM (Fatima et al., 2018). Such an allocation causes substantial congestion on the PM. Hence, this subsection proposes a hybrid VMP algorithm, HPSOLF-FPO, to overcome the local optima problem. The proposed algorithm improves the optimal solution discovery process, as the FPO algorithm is capable of updating solutions in the search space to continuously valued positions. The hybrid algorithm accomplishes three objectives. First, it achieves the required exploitation and exploration capabilities. Second, it enhances the search accuracy. Finally, it increases the probability of finding the global best solution to the problem. The algorithm combines the advantages of PSO local search with FPO global search. This overcomes the weakness of the global search capability of PSO and PSOLF, guaranteeing better placement decisions. The hybrid algorithm consists of three principal operations: population initialization, population evaluation, and termination.

• **Population initialization** As for swarm initialization (‘The PSOLF-based VMP algorithm stages’), the *N*_*swarm*_ particles are derived from the placement matrix 𝕄. The initial particle position and velocity are set, along with the particle fitness functions, particle local best *P*_*l*−*best*_ and the swarm global best *P*_*g*−*best*_. During each iteration, the equations used to update the particle velocities are determined by the value of *β* ∈ [0,1], where the switching probability *β* is used in place of the constant switching value 0.5, to select between Lévy flight equations or basic PSO equations. The use of *β* improves results. Furthermore, *β* can be controlled and changed for different experiments. As for pollen initialization (‘The FPO-based VMP algorithm stages’), the pollen grains that represent the possible solutions *S*_*i*_ are derived from the placement matrix 𝕄. The initial pollen parameters are set: position *S*_*i*_, fitness function, new solution *S*_*u*_ and best fitness value ${g}_{best}^{\ast }$. Consequently, all possible solutions represented by the *N*_*swarm*_ particles of the PSO module have corresponding pollen grains in the FPO module.
• **Population evaluation.** Across successive iterations, the population is evaluated across three modules: the PSO module, the FPO module, and the update module. During each iteration, the PSO module will compute all updated particle parameters. However, the particle parameters are not updated. Instead, the updated parameters are moved to the update module. In addition, the new particle positions *P*_*u*_ are input to the FPO module. According to the value of the switching probability, the FPO module performs either global pollination or local pollination. Consequently, all corresponding pollen parameters are updated. Within the update module, for each solution the updated position *S*_*u*_ generated by the FPO module is compared to the corresponding solution *P*_*u*_ generated by the PSO module. The lowest of the two is selected to update the corresponding position of both solutions in the PSO module and FPO module. Based on the updated position for each new solution, the remaining parameters are updated. They include the fitness function, *P*_*l*−*best*_, and *P*_*g*−*best*_. The merging of the updated solutions from both the PSO module and the FPO module successfully overcomes the local optima problem.
• **Termination** Population evaluation continues iteratively until the maximum number of iterations *Max*_*iter*_ is reached. Following the final iteration, *P*_*g*−*best*_ is selected as the optimal VMP solution.

The pseudo-code for the HPSOLF-FPO algorithm is presented below.


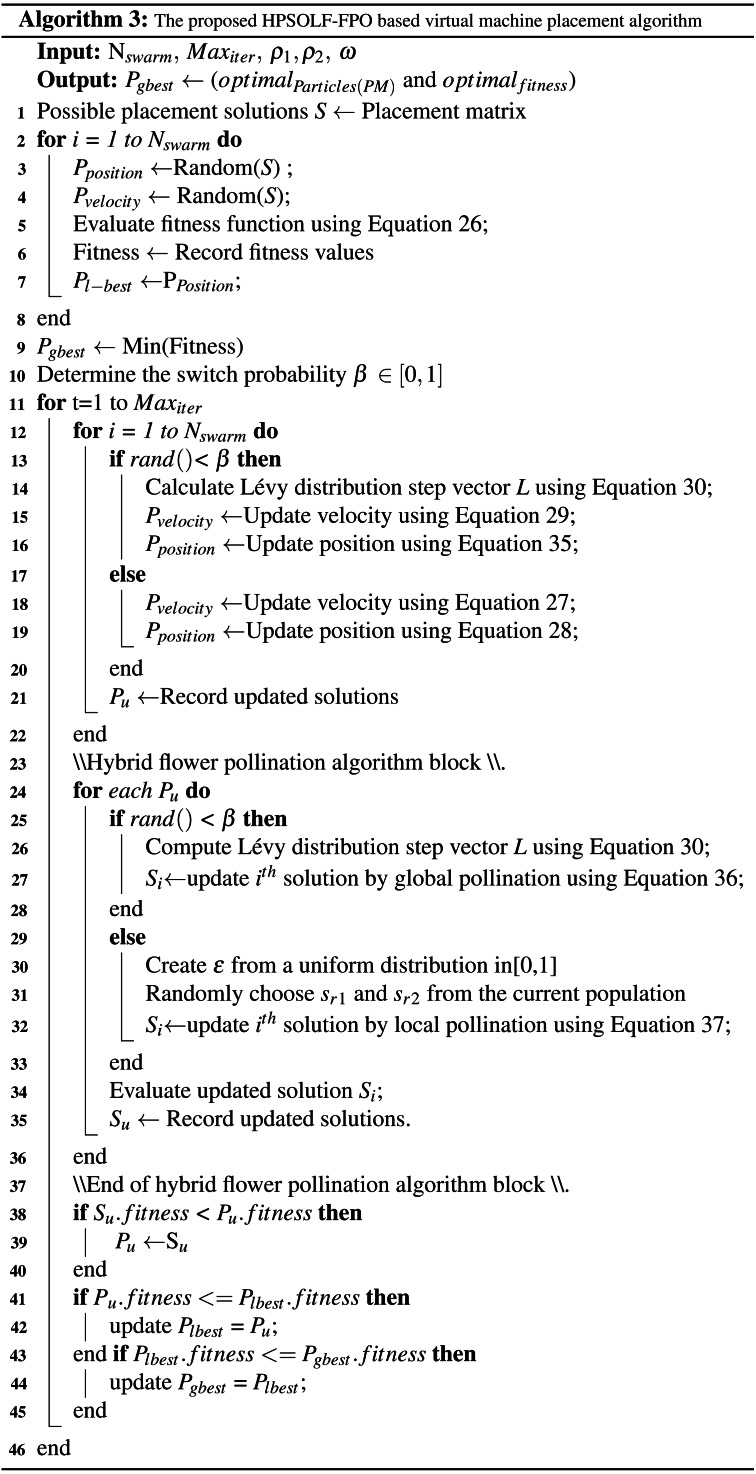


## Simulation evaluation

This section compares the results of experiments with the three proposed algorithms: PSOLF, FPO, and HPSOLF-FPO. The three algorithms are simulated using a MATLAB tool to generate 2000 VM requests. The remaining implementation parameters are listed in Table 1. The results from the three algorithms are compared to measure their performance in terms of placement time, power consumption, resource utilization (inferred from the number of active servers), and resource wastage.

**Table 1:** Parameter settings of the proposed algorithms.

Parameter name	Value
Number of iterations *Max*_*iter*_	100
Population size *N*_*pop*_	100
*ω*	2
*C*_1_	2
*C*_2_	2
*ρ*_1_	*U*[0–1]
*ρ*_2_	*U*[0–1]
Switch probability *β*	*U*[0–1]

### Fitness function performance

**Figure 4:**
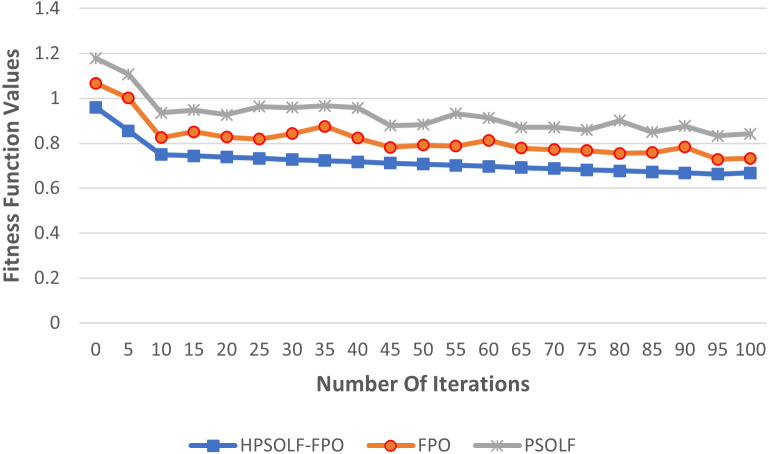
The fitness function values of the proposed VMP algorithms.

Figure 4 shows the values of the proposed fitness function against the experimental iterations for the three algorithms. The results show that the HPSOLF-FPO algorithm outperforms the other two algorithms in terms of the rate at which the fitness value decreases. In addition, the HPSOLF-FPO algorithm obtained the lowest fitness value in the smallest number of iterations.

### Placement time performance

**Figure 5:**
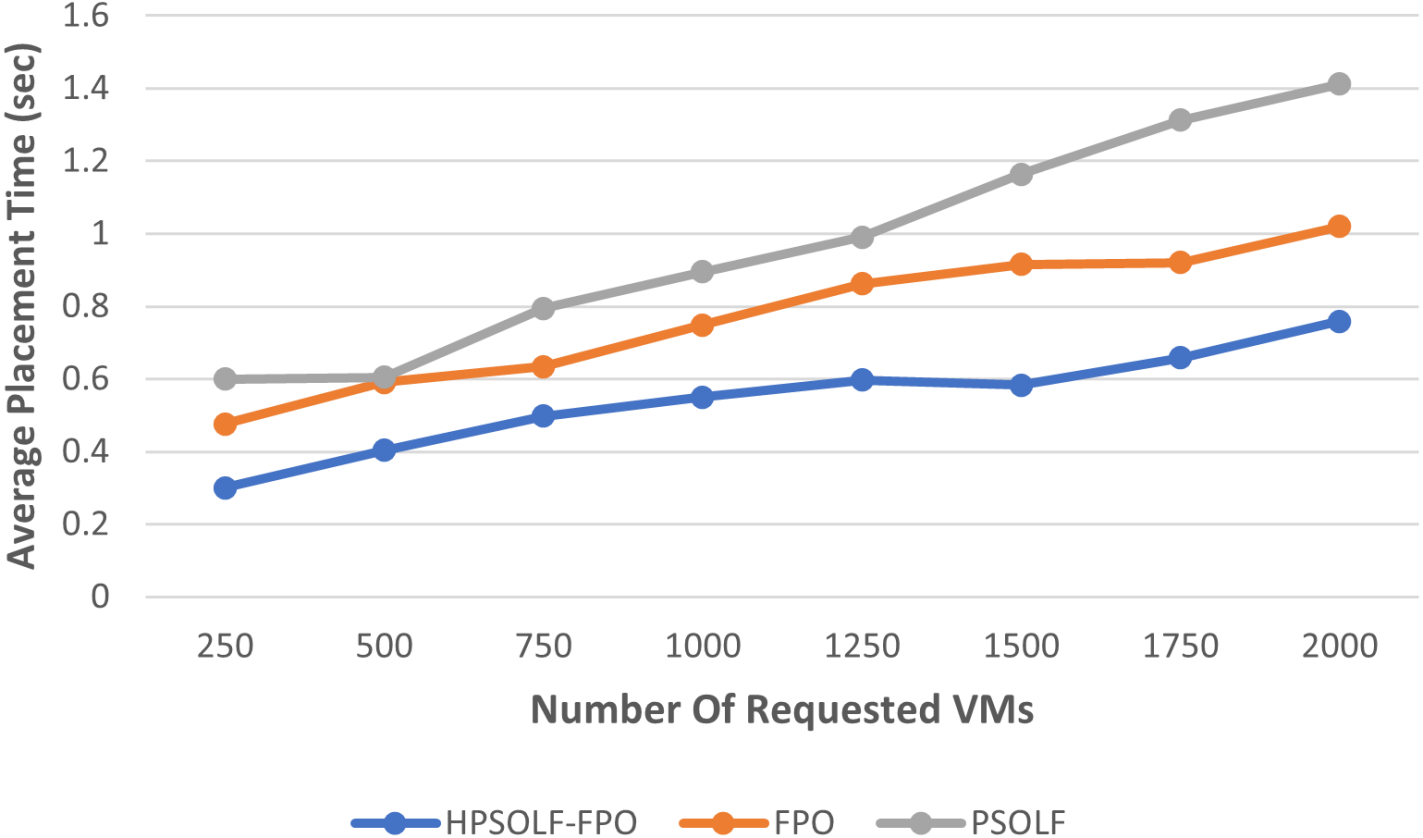
The average placement time against the number of requested VMs.

Figure 5 shows the average placement time for the three algorithms. The average placement times increase as the number of VM requests increase for each algorithm. However, the highest average placement times are generated by the PSO algorithm, while the lowest average are produced by the HPSOLF-FPO algorithm. Hence, the HPSOLF-FPO algorithm effectively reduces placement times.

### Power consumption performance

**Figure 6:**
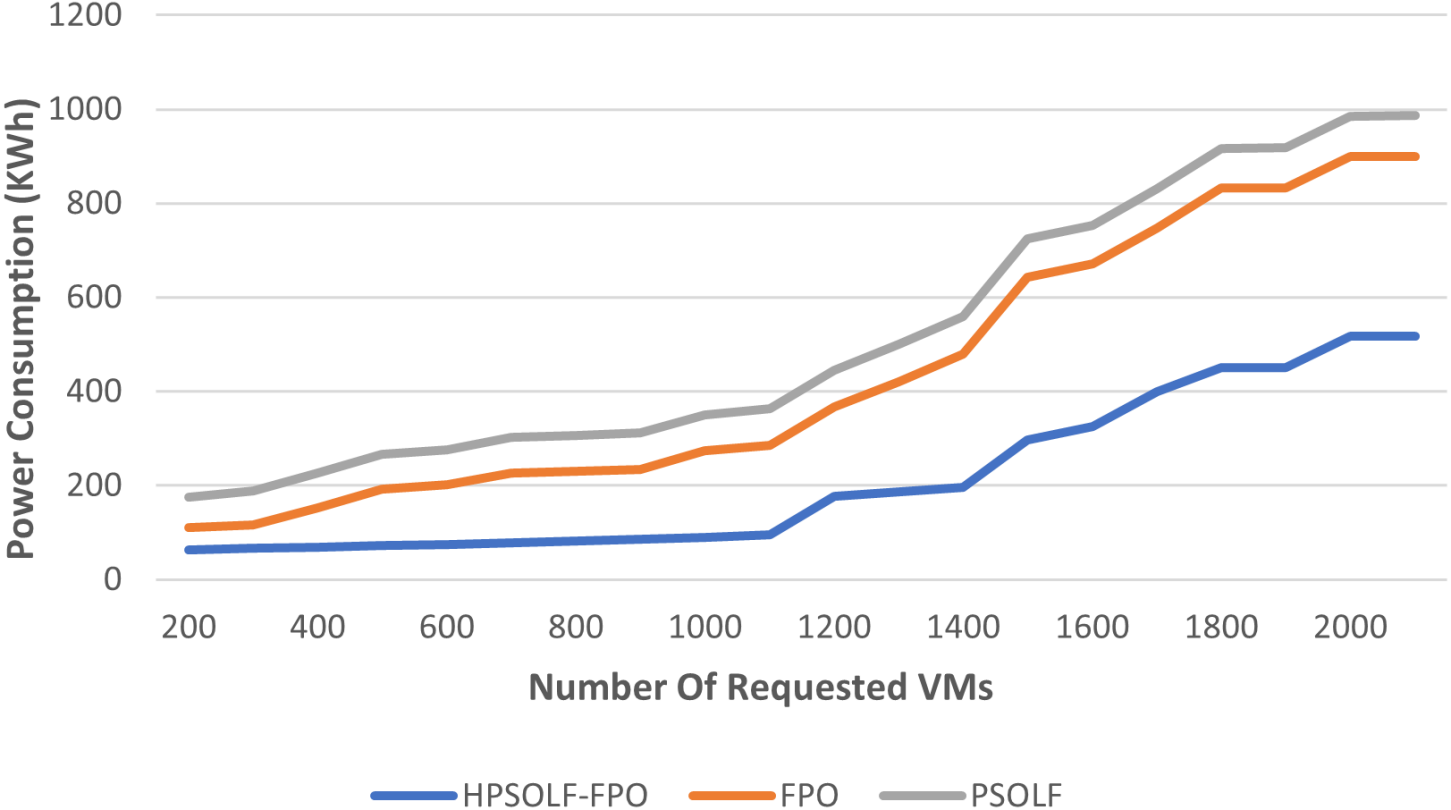
The power consumption against the number of requested VMs.

The total power consumption of all active servers for the three proposed algorithms is shown in Fig. 6. Power consumption increases as the number of VM requests increases for all algorithms. However, as for the number of requested VMs, the HPSOLF-FPO algorithm always has lower power consumption than the other algorithms. The placement strategy when using the PSO algorithm produces the highest power consumption.

### Resource utilization performance

**Figure 7:**
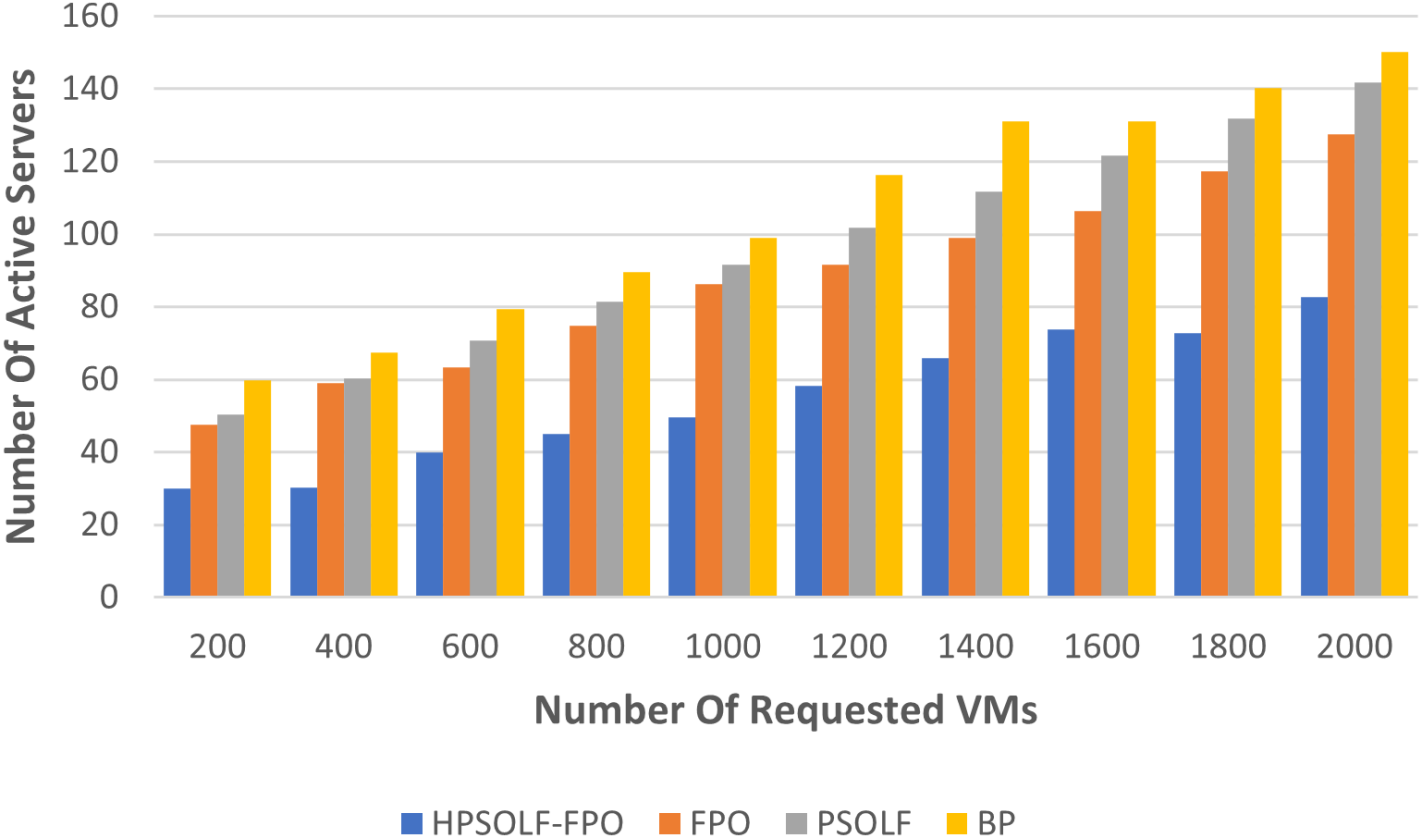
Active servers against the number of requested VMs.

To evaluate the performance of the algorithms in term of resource wastage, two different experiments were performed. The first experiment used each of the three algorithms in addition to a best-fit bin packing (BP) strategy. During the experiment the number of active servers was used to indicate the utilization rate of the PMs. Figure 7 shows the experimental results comparing all algorithms when using the same number of user requests. The three optimization algorithms outperform the best-fit bin packing strategy. The HPSOLF-FPO algorithm outperforms all other algorithms and has the minimum number of active servers. Hence, it effectively maximizes resource utilization in the cloud DC.

**Figure 8:**
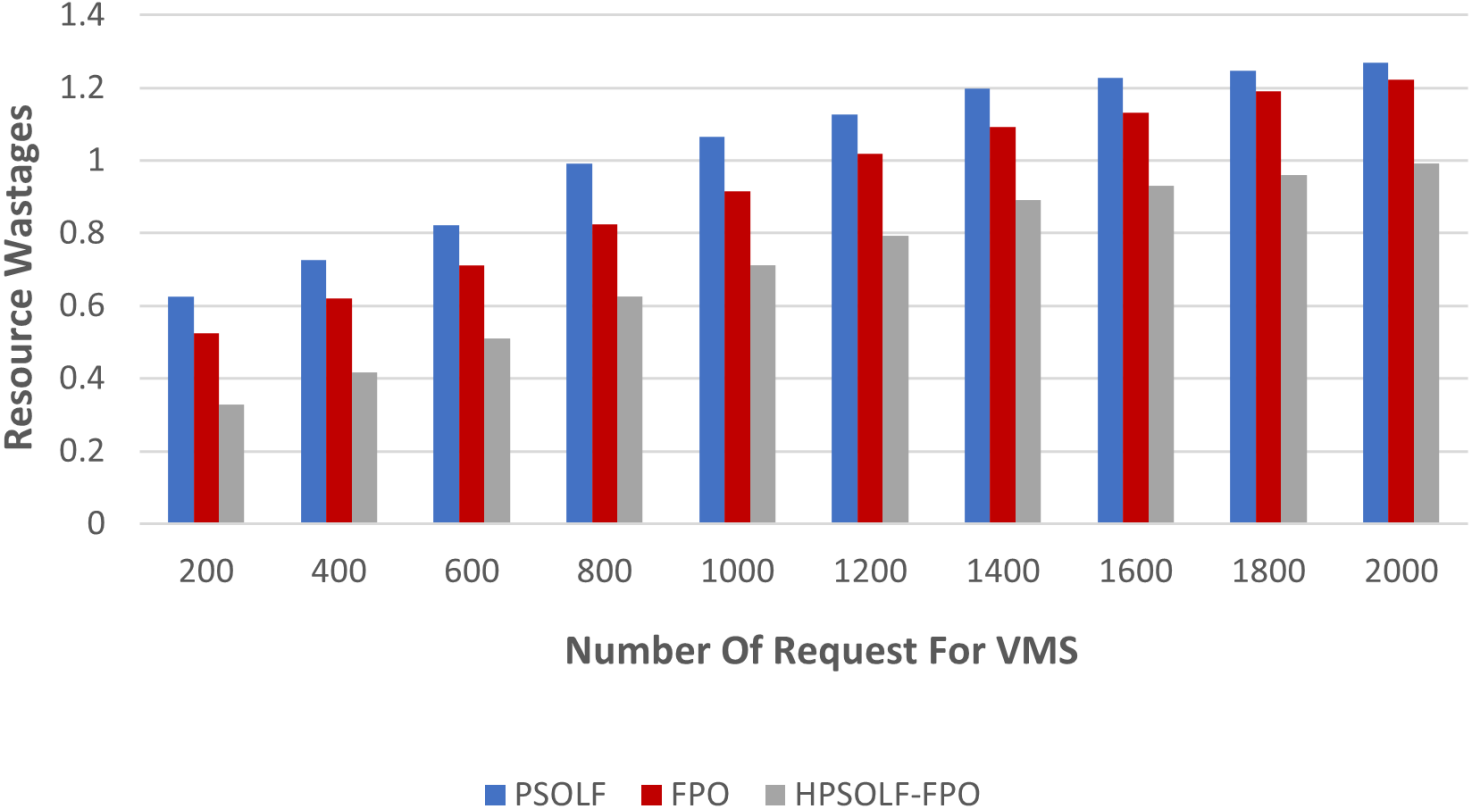
Resource wastage against the number of requested VMs.

The second experiment was performed using the three optimization algorithms to measure resource wastage. Figure 8 demonstrates the results. It can be observed that, with an increasing number of VM requests, the HPSOLF-FPO algorithm wastes fewer resources than the PSOLF and FPO algorithms. These results are obtained due to the decision making process of the HPSOLF-FPO algorithm which accounts for available remaining resources and uses them in a balanced manner to achieve minimum resource wastage.

## Conclusion and future work

This paper presented an efficient multi-objective VMP strategy for the cloud computing environment. The proposed model aims to determine the optimal VMP solution among all possible VMP solutions. The HPSOLF-FPO algorithm was developed to combine the exploration capabilities of FPO with the exploitation capabilities of PSO. The algorithm can move between FPO and PSO as needed. When trapped in local optima, the algorithm utilizes FPO to move away. As the local optima disappear, the algorithm returns to using PSO to improve its ability to obtain an optimal solution. The optimal VMP solution was evaluated based on a fitness function that combines the values of three criteria: the total placement time of requested VMs, power consumption, and resource wastage in the cloud DC. The HPSOLF-FPO, PSOLF, and FPO algorithms were evaluated based on the proposed fitness function. The experimental results of the simulation evaluations showed that the proposed HPSOLF-FPO algorithm is more efficient than the PSOLF or FPO algorithms. Furthermore, when the server utilization was measured, the HPSOLF-FPO algorithm outperformed the bin packing best-fit strategy. In future work, the proposed algorithms may be used to fulfil additional VMP objectives such as load balancing, live migration, and cost minimization. In addition, the proposed algorithm in combination with machine learning techniques will be employed to serve real-time or non-real-time tasks in a cloud computing environment.

## Supplemental Information

10.7717/peerj-cs.834/supp-1 Supplemental Information 1 Source Code The raw data contains the implementation of three proposed algorithms: PSO, FPO, and a hybrid PSO-FPO. These algorithms solve the virtual machine placement problem in cloud data centers.

## Additional Information and Declarations

### Data Availability

The following information was supplied regarding data availability: The raw data and code are available in the Supplemental File.
